# Cardiorespiratory fitness: a comparison between children with renal transplantation and children with congenital solitary functioning kidney

**DOI:** 10.1186/s13052-016-0299-7

**Published:** 2016-10-06

**Authors:** Riccardo Lubrano, Giancarlo Tancredi, Raffaele Falsaperla, Marco Elli

**Affiliations:** 1 Pediatric Department, Pediatric Nephrology Unit, Sapienza University of Rome, Rome, Italy; 2 General Pediatrics and Pediatric Acute and Emergency Unit, Policlinico-Vittorio-Emanuele University Hospital, Catania, Italy; 3 DIBIC-Biomedical and Clinic Science Department, “Luigi Sacco”, the University of Milan-VMS Vialba Medical School, Milan, Italy; 4 Servizio di Nefrologia Pediatrica, Dipartimento di Pediatria, Sapienza Università di Roma, Viale Regina Elena 324, 00161 Roma, Italia

**Keywords:** VO2 max, Cardiorespiratory fitness, Chronic renal failure, Child, Physical activity, Renal transplant, Solitary functioning kidney

## Abstract

Children with end-stage renal disease are known to have a cardiorespiratory fitness significantly reduced. This is considered to be an independent index predictive of mortality mainly due to cardiovascular accidents. The effects of renal transplantation on cardiorespiratory fitness are incompletely known. We compared the maximal oxygen uptake (VO2 max) of children with a functioning renal transplant with that of children with congenital solitary functioning kidney, taking into consideration also the amount of weekly sport activity.

Dear Editor,

Patients with chronic renal failure (CRF) tend to reduce their weekly amount of physical activity, with negative effects on cardiorespiratory fitness and quality of life. After renal transplant the metabolic deficits induced by CRF are partially recovered and cardiorespiratory fitness improves. As we previously reported cardiorespiratory fitness of transplanted children practicing sports for more than 3 h per week is similar to normal controls exercising less that 3 h [1]. On the contrary, cardiorespiratory fitness of children with a congenital solitary functioning kidney is similar to normal controls exercising for a comparable number of hours [2].

We measured the aerobic capacity in relation with weekly amount of physical activity and glomerular filtration rate (GFR), comparing a group of children with a congenital solitary functioning kidney (cSFK) and a group of children with a functioning renal transplant (Tx).

A standardized pediatric questionnaire was administered to all children for investigating the time dedicated weekly to physical activity [3]. On the basis of the questionnaire, the children were divided into inadequately active (<3 h of physical activity per week) and adequately active (>3 h of physical activity per week).

In the cSFK group we enrolled 30 patients: 15 exercising more than 3 h/week (cSFK>3) and 15 less than 3 h/week (cSFK<3). The Tx group was formed with 20 children, 10 exercising more than 3 h/week (Tx>3) and 10 less than 3 h/week (Tx<3). In all patients, transplant had been performed 6 or more years previously, following a dialysis treatment never exceeding on year. All received triple immunosuppressive therapy: 12 with tacrolimus and 8 with cyclosporine.

Maximal oxygen uptake (VO2 max) was measured during a maximal incremental exercise on a treadmill (Bruce protocol) consisting of sequential increase in speed and slope every 3 min until exhaustion (breathlessness and leg muscle pain) and/or heart rate ≥85 % of maximum (calculated with the formula 220 – age in years). During the exercise the subjects were connected by face mask to a breath-by-breath analyser of O2 to measure the oxygen consumption (VO2). Maximal oxygen uptake (VO2 max) was defined as the highest level of VO2 reached during the maximal exercise test expressed as VO2 max/kg (ml/min/kg).

The glomerular filtration rate (ml/min/1.73mq) was calculated with the creatinine clearance. Informed consent was obtained from both parents. The protocol conforms to the guidelines of the Declaration of Helsinki and was approved by the ethical committee of the involved institution.

The children in all groups were comparable for age (years: Tx>3 12.67 ± 3.56; Tx<3 13.90 ± 1.20; cSFK>3 14.18 ± 5.29; cSFK<3 13,5 ± 4.76; p NS). GFR was also similar in all groups (GFR ml/min/1.73 mq: Tx>3 90.65 ± 22.52; Tx<3 92.02 ± 21.18; cSFK>3 99.15 ± 30.63; cSFK<3 101,02 ± 40.12; p NS).

VO2 max in Tx and cSFK was significantly higher in those practicing sport for more than 3 h per week (Table 1). Children with a congenital solitary functioning kidney had level of VO2 max consistently and significantly higher than transplanted patients (Table 1). There was no significant correlation between VO2 max and GFR (VO2 max = 26.08 + 0.006; GFR R^2 = − 0.07).

**Table 1:** VO2 max/kg in the four groups of the study

	Tx>3	Tx<3	cSFK>3	cSFK<3
VO2 max/kg ml/min/kg	28.99 ± 1.15	23.22 ± 1.23	46.12 ± 1.09	38.55 ± 1.97

Tx>3 vs Tx<3 *p* < 0.003; cSFK>3 vs cSFK<3 *p* < 0.01; Tx>3 vs cSFK>3 *p* < 0.016; Tx<3 vs cSFK<3 *p* < 0.001; Tx<3 vs cSFK>3 *p* < 0.004; Tx>3 vs cSFK<3 *p* < 0.001

Our findings show that not only congenital solitary functioning kidney (cSFK), but also transplanted children with regular physical activity exceeding three hours weekly achieve higher levels of VO2 max. Adequate and regular physical exercise proves therefore beneficial in transplanted children improving their ability to cope with the increased metabolic request of physical stress and therefore reducing the risk of mortality from cardiovascular disease [4].

VO2 max in transplanted children is consistently lower than single kidney patients with comparable physical activity. This may be due in part to the neuromuscular, metabolic, and cardiopulmonary deficits acquired during the exposure to uremic intoxication before transplant [5], that a functioning graft can improve but not reverse completely. A combination of early transplant and prompt resumption of controlled adequate physical exercise post-transplant is likely to improve further the cardiorespiratory fitness in these patients, with the known benefits on cardiovascular risk and mortality.

## Abbreviations

3 h/week: 3 hours a week
CRF: Chronic renal failure
cSFK: Congenital solitary functioning kidney
GFR: Glomerular filtration rate
Tx: Transplanted children
VO2 max: Maximal oxygen uptake
